# When age tips the balance: A dual mechanism affecting hemispheric specialization for language

**DOI:** 10.1162/IMAG.a.63

**Published:** 2025-07-02

**Authors:** Elise Roger, Loïc Labache, Noah Hamlin, Jordanna Kruse, Monica Baciu, Gaelle E. Doucet

**Affiliations:** Institut Universitaire de Gériatrie de Montréal, Communication and Aging Lab, Montreal, Quebec, Canada; Faculty of Medicine, University of Montreal, Montreal, Quebec, Canada; Univ. Grenoble Alpes, Univ. Savoie Mont Blanc, CNRS, LPNC, Grenoble, France; Department of Psychology, Yale University, New Haven, CT, United States; Department of Psychiatry, Brain Health Institute, Rutgers University, Piscataway, NJ, United States; Institute for Human Neuroscience, Boys Town National Research Hospital, Omaha, NE, United States; Department of Pharmacology and Neuroscience, Creighton University School of Medicine, Omaha, NE, United States; Center for Pediatric Brain Health, Boys Town National Research Hospital, Omaha, NE, United States

**Keywords:** language, memory, lateralization, lifespan, gradient, aging

## Abstract

Aging is accompanied by changes in brain architecture that alter the lateralization of functional networks. In this study, we examined how hemispheric specialization changes across the adult lifespan by analyzing resting-state fMRI and structural MRI data from 728 typical adults aged 18 to 88 years. Using the Language-and-Memory Network atlas, we quantified regional asymmetries in functional connectivity along the cortex’s principal gradient, and normalized regional volumes across 37 bilateral regions. We identified two distinct age-related asymmetry trajectories: one pattern revealed a bilateralization of language-dominant regions, while the other showed increasing leftward specialization in multimodal regions associated with memory and language. These opposing patterns emerged around midlife and were linked to performance in language production tasks. By integrating connectivity gradients, structural asymmetries, and behavioral data, our findings provide new evidence for a dual mechanism reshaping functional brain lateralization with age and demonstrate the utility of resting-state metrics in tracking these shifts.

## 1 Introduction

The human brain exhibits marked hemispheric specialization, translated by differences in structure and function between the left and right hemispheres, which underpin key cognitive abilities such as language (Hervé et al., 2013). While structural asymmetries refer to anatomical differences (e.g., in cortical thickness or regional volume), functional lateralization describes the unequal distribution of cognitive functions, such as language processing, across hemispheres. Both forms of asymmetry emerge early in life, as evidenced by prenatal imaging studies showing perisylvian asymmetries by 26 gestational weeks (Kasprian et al., 2011), and become increasingly pronounced during development (Abu-Rustum et al., 2013; Kong et al., 2018).

Language lateralization, a fundamental characteristic of human brain organization (Güntürkün & Ocklenburg, 2017), traditionally emphasizes the left hemisphere’s dominance for core language functions (Tzourio-Mazoyer et al., 2017). However, neuroimaging has revealed that language processing engages a broader set of regions beyond the classical Broca’s and Wernicke’s areas. These include transmodal associative hubs that support the integration of linguistic, mnemonic, and executive information (Braga et al., 2020; Labache et al., 2019, 2020; Roger et al., 2022; Salvo et al., 2024). This extended language network includes areas in the anterior temporal lobe, medial frontal cortex, and posterior cingulate, as well as subcortical and cerebellar structures (Wolna et al., 2025), forming a distributed, functionally integrated system.

Several approaches have been used to define the architecture of the language network. A key distinction lies between group-level averaging and individual-subject functional localization. Fedorenko and colleagues’ work demonstrates that language-selective areas can be identified with high reliability across individuals using dedicated language localizers (Fedorenko & Thompson-Schill, 2014; Fedorenko et al., 2010). More recently, individualized functional connectomics has enabled the identification of the language network even in resting-state and non-related task data (Shain & Fedorenko, 2025). These efforts culminated in a probabilistic atlas of the language network from over 800 individuals (Lipkin et al., 2022), highlighting robust left-lateralized frontotemporal patterns comparable with previous work (Labache et al., 2019) and meta-analysis (Price, 2012).

Despite advances in mapping the language connectome, age-related changes in functional lateralization remain poorly understood (Baciu & Roger, 2024). While some studies suggest a decline in lateralization with aging (Festini et al., 2018), others report compensatory bilateralization or preserved asymmetries in certain regions (Turner & Spreng, 2015). Moreover, language lateralization may evolve differently across subcomponents of the language system, especially in regions supporting multimodal integration or memory (Roger et al., 2022).

To address this gap, we investigate how functional asymmetries in the extended language network evolve across the adult lifespan. We use resting-state fMRI to derive individual-level measures of lateralization. Resting-state functional connectivity reliably reflects the architecture of task-based language networks (Braga et al., 2020; Cole et al., 2014, 2016; Ji et al., 2019) and provides a window into intrinsic brain organization. Our focus is on the principal gradient of connectivity (G1), a macroscale functional gradient that organizes the cortex from unimodal to heteromodal regions (Margulies et al., 2016). Prior work has shown that G1 differs between hemispheres and correlates with language dominance (Labache, Ge, et al., 2023; Margulies et al., 2016).

The goal of this study is to elucidate the mechanisms by which age-related changes in brain asymmetry impact language processing and cognitive functions. Our innovative approach combines the analysis of resting-state functional connectivity (macroscale functional gradient G1) with advanced statistical modeling to provide a comprehensive view of how the aging brain adapts its functional architecture. We opted for the Language-and-Memory Network due to its comprehensive ability to capture the nuanced dynamics of language in conjunction with other cognitive processes (Roger et al., 2020). The Language-and-Memory Network integrates regions specialized in language processing with areas concurrently involved in language and advanced cognitive functions, such as memory and executive processes. Importantly, these heteromodal regions may undergo significant functional changes with aging. To model the functional trajectories over an age range from 18 to 88 years, we applied the Generalized Additive Mixed Models (GAMMs) technique, which has been previously used in structural MRI studies (Roe et al., 2021, 2023). This allowed us to classify Language-and-Memory Network regions based on their asymmetry patterns at rest throughout normal aging. Furthermore, we also explored how these asymmetry changes were related to cognitive performance measured during various language tasks. To this end, we used Canonical Correlation Analysis (CCA) to assess how age impacted asymmetries in the language network across multimodal data, including anatomy, function, and cognitive performances.

The study’s findings will advance our understanding of how normal aging impacts complex brain networks. This research aligns with the rising global emphasis on geroscience, which aims to elucidate the biological mechanisms of aging and foster strategies for maintaining health in older adults. By identifying potential biomarkers for early detection of age-related cognitive decline, it supports the development of targeted interventions to preserve or improve cognitive health. It may pave the way for personalized neurorehabilitation approaches by providing valuable insights into individual differences in brain asymmetry and cognitive function. Given the urgent need to address cognitive decline and enhance cognitive longevity in an aging population, these insights are both timely and crucial.

## 2 Methods

### 2.1 Database demographics

**Fig. 1.**
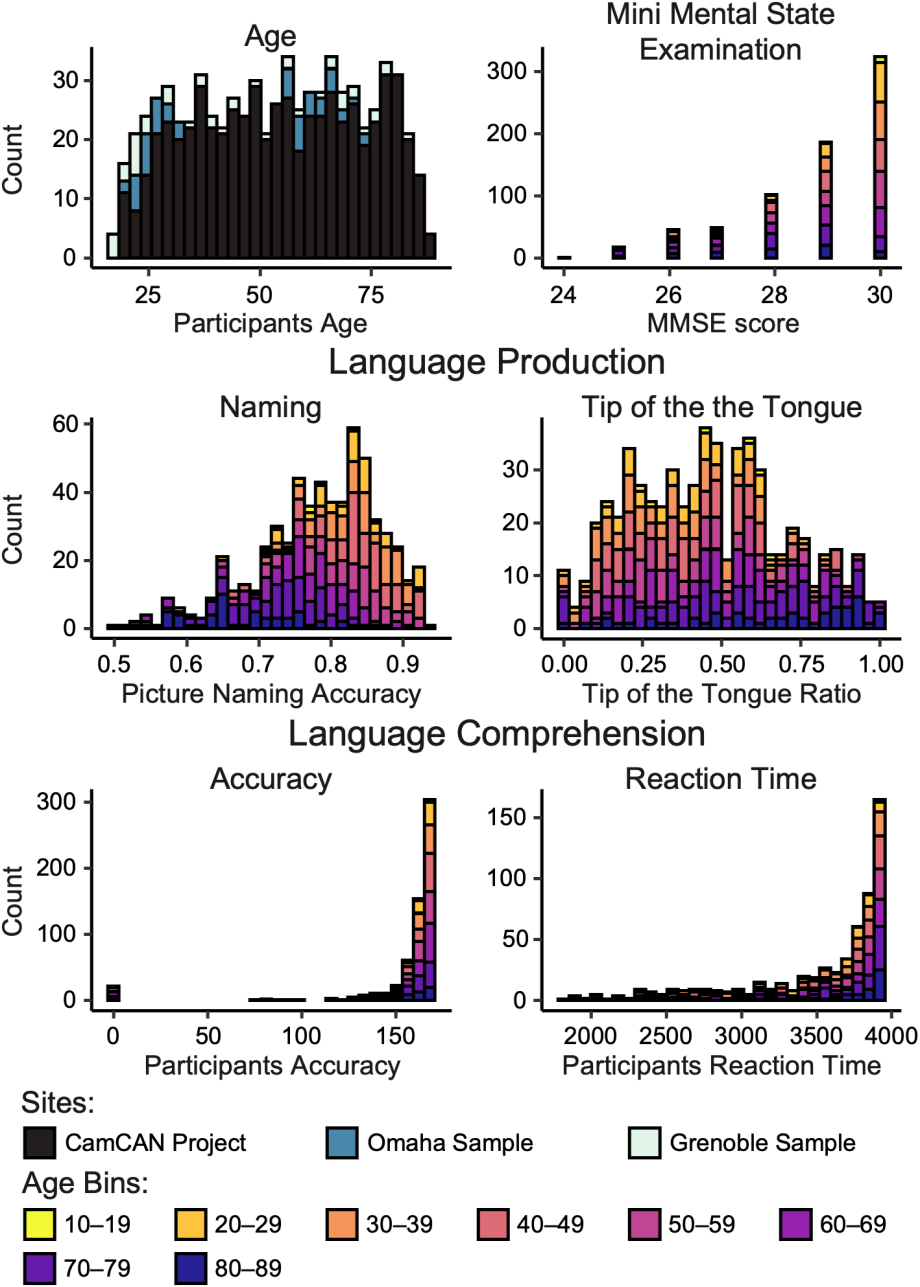
Age and behavioral performance stacked distributions. The behavioral tests assess various cognitive functions associated with language: word production, lexical access/retrieval abilities (picture naming accuracy and tip of the tongue ratio), and semantic and syntactic comprehension abilities (accuracy and reaction time). A description of the behavioral variables is available as Supplementary Materials in the article by West et al. (2022). Reaction time and tip of the tongue performance were inverted, so all scores close to zero represent worse performances. Stacked histograms for age and MMSE include 728 participants. Language Production and Language Comprehension stacked histograms include 554 participants of the CamCAN database only due to a lack of behavioral data for other participants.

The study sample comprised 3 datasets, accumulating 728 healthy adults (371 women) from 18 to 88 years old (*μ* = 52.84 years, SD = 19.19 years, Fig. 1). Included participants had resting-state (rs) fMRI and structural MRI from a 3T scanner, meeting criteria of passing quality checks (*fmriprep* QC reports) and exhibiting no confirmed neurological or psychiatric pathologies.

The larger sample, the Cambridge Centre for Ageing and Neuroscience Project (Shafto et al., 2014) (CamCAN Project: www.mrc-cbu.cam.ac.uk), included 627 participants (316 women). Structural MRI data were acquired on a 3T Siemens TIM Trio scanner with a 32-channel head coil, using a T1-weighted, 3D MPRAGE sequence with the following parameters: repetition time (TR)/echo time (TE)/inversion time (TI) = 2250/2.99/900 ms, voxel size = 1 mm isotropic, flip angle = 9°, field of view (FOV) = 256 × 240 × 192 mm^3^, duration of acquisition: 4 min 32 s. For resting-state fMRI scans, participants rested with their eyes closed for 8 min 40 s. Two hundred and sixty-one brain volumes were acquired using a gradient echo planar imaging sequence (EPI, 32 axial slices, 3.7 mm thickness, TR = 2.0 s, TE = 30 ms, flip angle = 78°, FOV = 192 × 192 mm^2^, voxel size = 3 × 3 × 4.44 mm^3^). Further recruitment information and the acquisition parameters have been described elsewhere (Taylor et al., 2017). The sample mean age was 54.28 years (SD = 18.61 years). Participants’ handedness was defined based on the manual preference strength assessed with the Edinburgh inventory (Oldfield, 1971): participants with a score below 30 were considered left-handers (Hervé et al., 2006; Papadatou-Pastou et al., 2020), right-handers otherwise. The sample contained 56 left-handed participants (32 women). CamCAN funding was provided by the UK Biotechnology and Biological Sciences Research Council (grant number BB/H008217/1), with support from the UK Medical Research Council and the University of Cambridge, UK.

The second sample was collected in Omaha, NE, USA, and included 54 participants (31 women). The acquisition parameters are fully described in Doucet et al. (2022). Briefly, participants were scanned on a 3T Siemens Prisma scanner using a 64-channel head coil. Structural images were acquired using a T1-weighted, 3D magnetization-prepared rapid gradient-echo (MPRAGE) sequence with the following parameters: TR = 2400 ms, TE = 2.22 ms, FOV: 256 × 256 mm, matrix size: 320 × 320, 0.8 mm isotropic resolution, TI = 1000 ms, 8 degree-flip angle, bandwidth = 220 Hz/Pixel, echo spacing = 7.5 ms, in-plane acceleration GRAPPA (GeneRalized Autocalibrating Partial Parallel Acquisition) factor 2, total acquisition time ~7 min. Participants also completed a resting-state fMRI scan (eyes open). Scans were performed using a multi-band T2* sequence with the following acquisition parameters: TR = 800 ms, TE = 37 ms, voxel size = 2 × 2 × 2 mm^3^, echo spacing 0.58 ms, bandwidth = 2290 Hz/Pixel, number of axial slices = 72, multi-band acceleration factor = 8, 460 volumes. The sample mean age was 44.13 years (SD = 19.07 years). Participants’ handedness was self-reported: the sample contained seven left-handed participants (three women). The Institutional Review Board for Research with Human Subjects approved the study at Boys Town National Research Hospital. Each participant provided written informed consent and completed the same protocol.

The third sample was collected in Grenoble, France, and included 47 participants (24 women). T1‐weighted high‐resolution three‐dimensional anatomical volumes (T1TFE, 128 sagittal slices, 1.37 mm thickness, FOV = 224 × 256 mm^2^, 0.8 mm isotropic resolution) were acquired for each participant by using a whole‐body 3T MR Philips imager (Achieva 3.0 T TX Philips, Philips Medical Systems, Best, NL) with a 32-channel head coil. For resting-state fMRI scans, 400 volumes were acquired using a gradient echo planar imaging sequence (FEEPI, 36 axial slices, 3.5 mm thickness, TR = 2.0 s, TE = 30 ms, flip angle = 75°, FOV = 192 × 192 mm^2^, voxel size = 2 × 2 × 2 mm^3^). Participants were asked to lie down in the scanner with eyes open on a central cross for the duration of the acquisition period (13 min 20 s). The sample mean age was 43.57 years (SD = 21.92 years). Participants’ handedness was self-reported: the sample contained two left-handed participants (one woman). The ethics committee of the Grenoble Alpes University Hospital approved data collection (CPP 09-CHUG-14; MS-14-102).

The Supplementary Materials (Comparative Tables of Database Acquisition Parameters section) provides comparative tables of database acquisition parameters.

We used the whole age range of the sample (*n* = 728, 18–88 years) to model the asymmetry trajectories further throughout the lifespan. By merging the CamCAN cohort with Grenoble and Omaha samples, we expanded our age coverage from 18 to 88 years old, addressing the lack of young adults in the CamCAN cohort (as depicted in Fig. 1), and making the age distribution uniform, allowing a more reliable analysis.

Although the three datasets were acquired on different MRI scanners with varying acquisition parameters, several precautions were taken to ensure the validity of combining them into a unified analysis. First, all imaging data were preprocessed using the same standardized pipeline (*fmriprep*), including spatial normalization to MNI space and uniform regression of nuisance variables. Second, site was explicitly modeled as a covariate of no interest in all statistical analyses using Generalized Additive Mixed Models, a flexible framework well suited for accounting for both linear and nonlinear inter-site differences (Roe et al., 2021, 2023). Third, a validation analysis comparing asymmetry trajectory classifications between the CamCAN-only sample and the full combined sample revealed a high Sørensen–Dice index (*SDI* = 0.92; Dice, 1945; T. Sørensen, 1948), confirming the robustness and reproducibility of the results across cohorts. Furthermore, combining datasets from different institutions enhances the generalizability of findings (Thompson et al., 2020). Large-scale consortia such as ENIGMA, which aggregate data across numerous scanners and sites, have demonstrated the value and feasibility of such approaches (Thompson et al., 2020). Importantly, this study uses a cross-sectional design, and thus age-related patterns reflect inter-individual differences rather than within-subject longitudinal change.

### 2.2 Cognitive assessment of participants

For all 728 participants, we checked the Mini Mental State Examination (MMSE) scores to ensure that the general cognitive functioning of our sample remained within the expected range (*Q_1_* = 28, *Q_3_* = 30).

Among the three cohorts in our study, only the CamCAN cohort underwent an extensive set of behavioral assessments, resulting in cognitive data available for a specific sub-sample of 554 participants. These assessments, conducted outside the MRI scanner, are detailed in the literature (Samu et al., 2017; Taylor et al., 2017). We limited our analyses to language skill assessments only (Fig. 1). We chose language-related measures because of their effectiveness in assessing diverse language-related aspects, encompassing word production, lexical access, and word retrieval (evaluated via picture naming accuracy and the tip-of-the-tongue ratio), as well as the understanding of semantics and syntax (measured through accuracy and reaction time). Further comprehensive descriptions of these behavioral variables are available in the Supplementary Materials provided by West et al. (2022).

### 2.3 MRI data preprocessing

The neuroimaging data were formatted following the BIDS standard (Gorgolewski et al., 2016; Roger et al., 2020) (Brain Imaging Data Structure—http://bids.neuroimaging.io/) and then preprocessed using the *fMRIPrep* software (https://fmriprep.org/en/stable/; Esteban et al., 2019, 2020). *fMRIPrep* version 21.0.2 was run using its default processing pipeline in a containerized environment with singularity, ensuring computational reproducibility. The T1w preprocessing included skull stripping, tissue segmentation, and spatial normalization. T1-weighted images were corrected for intensity non-uniformity using N4BiasFieldCorrection (ANTs), skull-stripped with antsBrainExtraction.sh, and spatially normalized to the ICBM 152 Nonlinear Asymmetrical template (*MNI152NLin2009cAsym*) using nonlinear registration with ANTs. Preprocessing of the rs-fMRI data followed the consensus steps for functional images, including motion correction, slice timing correction, susceptibility distortion correction, coregistration, and spatial normalization. The rs-fMRI images were motion corrected using FSL’s MCFLIRT, slice-time corrected using AFNI’s 3dTshift, and coregistered to the T1w using boundary-based registration (*bbregister*). Susceptibility distortion correction was applied using fieldmap-less correction with SyN in ANTs. Functional images were then normalized to MNI space using the same ANTs transformations. The data were represented in the Montreal Neurological Institute (MNI) volumetric space. Finally, time series were extracted for each homotopic region of interest (described in the following subsection) using *Nilearn* (https://nilearn.github.io/) with nuisance parameter regression. Before time series extraction, data were spatially smoothed with a 6 mm FWHM Gaussian kernel and temporally filtered (0.01–0.1 Hz) to remove low-frequency drift and high-frequency noise. Confounding regression included cerebrospinal fluid and white matter signals and translation and rotation parameters for *x*, *y*, and *z* directions.

### 2.4 Language-and-memory network statistics

Our statistical analyses were based on the Language-and-Memory Network atlas, an extended language network encompassing language-specific areas and related memory regions (Roger et al., 2020). Briefly, the Language-and-Memory Network comprises 37 homotopic regions of interest. Among these 10 regions uniquely dedicated to the core supramodal language network (Labache et al., 2019), 19 supporting episodic memory (Spaniol et al., 2009) and 8 regions underpinning both language and episodic memory processes. The core language network corresponded to a set of heteromodal brain regions significantly involved, leftward asymmetrical across three language contrasts (listening to, reading, and producing sentences), and functionally connected (Labache et al., 2019). This functional asymmetry aligns with longstanding evidence of anatomical asymmetries in perisylvian cortex that are thought to form a structural basis for the evolution and development of language-related circuits (Hutsler, 2003; Kong et al., 2022; Meyer et al., 2014; Toga & Thompson, 2003; Tzourio-Mazoyer et al., 2018).

These anatomical asymmetries, including the early developing leftward expansion of the planum temporale and superior temporal sulcus, are evident from the 23^rd^ gestational week (Tzourio-Mazoyer et al., 2020) and are believed to result from developmental gradients in cortical neurogenesis and radial migration (Geschwind & Rakic, 2013; Rakic, 1988). While such asymmetries likely scaffold the emergence of left-lateralized language function (Achorn et al., 2025), they do not directly account for the specialization of higher-order linguistic processes. Recent neurocognitive models (Fedorenko & Thompson-Schill, 2014; Matchin & Hickok, 2020) emphasize that early perceptual and phonological operations localize to these structurally asymmetric regions, whereas syntactic and combinatorial computations are distributed across functionally lateralized fronto-temporal circuits. This dissociation supports the view that structural and functional asymmetries, although developmentally related, subserve distinct stages of language processing.

**Fig. 2.**
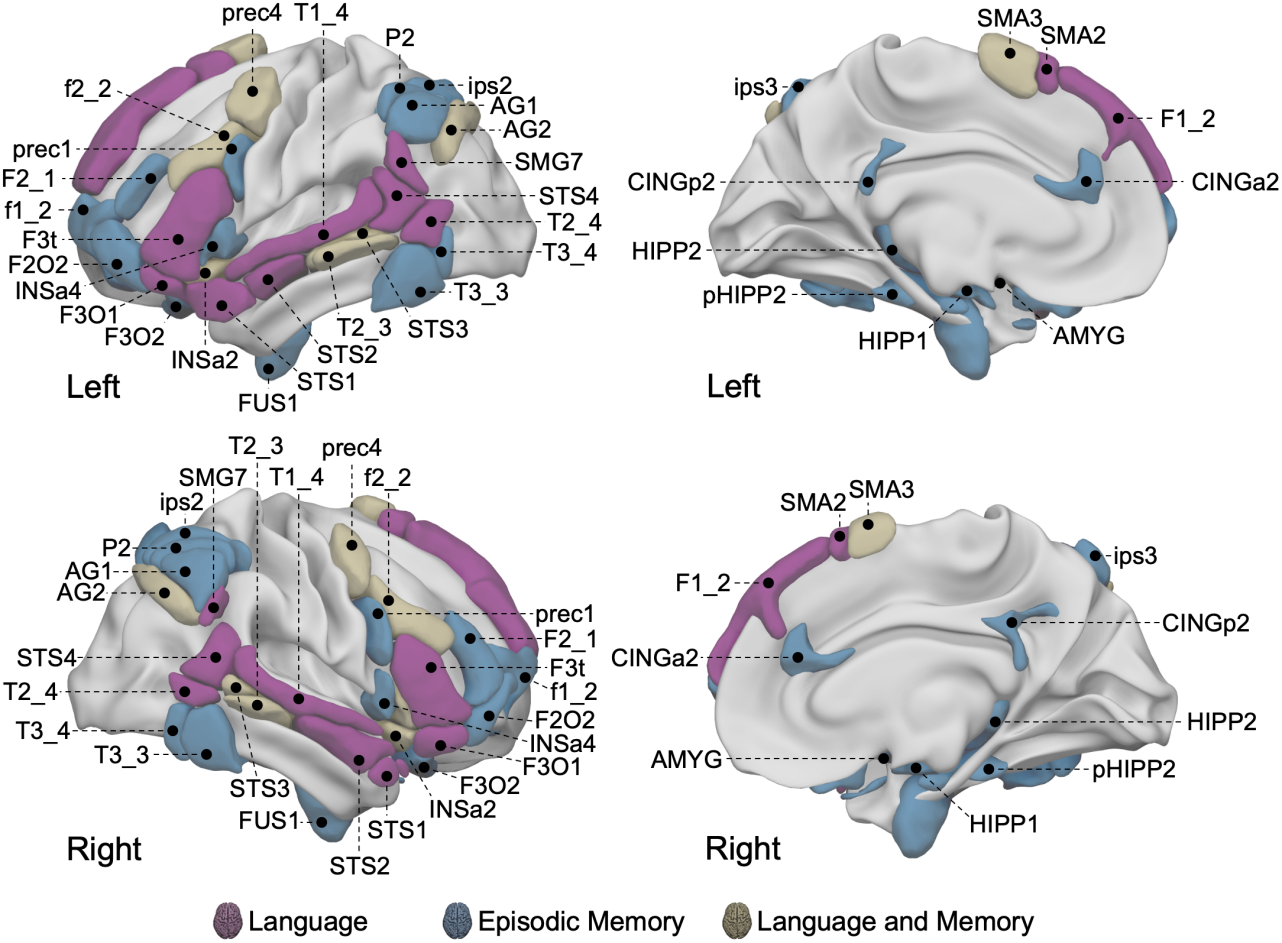
Locations of the 37 regions from the Language-and-Memory Network atlas in the left hemisphere and their homotopic counterparts in the right hemisphere (Roger et al., 2020). On the left: lateral view of the left (top row) and right (bottom row) hemisphere. On the right: medial view of the left (top row) and right (bottom row) hemisphere. The atlas is composed of 74 homotopic ROIs (37 in each hemisphere) reported by 2 task-fMRI studies, 1 cross-sectional study for language (Labache et al., 2019), and 1 meta-analysis for memory (Spaniol et al., 2009) and adapted to the atlas of intrinsic connectivity of homotopic areas coordinates (Joliot et al., 2015). Regions are rendered onto the 3D anatomical templates of the white matter surface of the left hemisphere in the MNI space with Surf Ice software (www.nitrc.org/projects/surfice/). Color code: purple, regions involved in language; blue, regions involved in episodic memory (encoding and retrieval); brown, regions involved in both language and memory. The anterior insula (3) (INSa3) is not visible on this render. See Supplementary Table S1 for the correspondences between the abbreviations and the full names of the Language-and-Memory Network regions.

Furthermore, the memory network was underpinned by areas that demonstrated strong activation patterns connected to episodic memory processes, such as encoding, effective recovery, and reminiscence. Figure 2 shows the Language-and-Memory Network in a brain rendering, and Supplementary Table S1 lists all the Language-and-Memory Network regions. It should be noted that the language atlas was based on the AICHA atlas, a functional brain homotopic atlas optimized for studying functional brain asymmetries (Joliot et al., 2015).

We computed two features characterizing the high-order Language-and-Memory Network regions (Roger et al., 2020) from the preprocessed neuroimaging data: the normalized volume and the first functional gradient (G1) reflecting the macroscale functional organization of the cortex (Margulies et al., 2016). The first gradient captures the most variance of the correlations matrices (20%, 22%, and 19% for CamCAN, Omaha’s, and Grenoble’s cohorts, respectively). It has been previously shown to accurately reflect the lateralization of the language network (Labache, Ge, et al., 2023).

### 2.5 Normalized volume

Tissue segmentation was performed on the preprocessed T1w using the *FreeSurfer* pipeline (Version 6.0.0; CentOS Linux 6.10.i386; Fischl et al., 2004). Briefly, the *FreeSurfer* segmentation process included the segmentation of the subcortical white matter and deep gray matter volumetric structures, intensity normalization, tessellation of the gray matter white matter boundary, automated topology correction, and surface deformation following intensity gradients to optimally place the gray/white and gray/cerebrospinal fluid borders at the location where the greatest shift in intensity defines the transition to the other tissue class. Structural volumes were normalized to total intracranial volume. Normalized volumes were extracted for each of the Language-and-Memory Network regions.

### 2.6 Connectivity embedding

Each participant’s values were obtained for the first functional gradient (G1). The gradients reflect participant connectivity matrices, reduced in their dimensionality through the approach of Margulies et al. (2016). Functional gradients reflect the topographical organization of the cortex in terms of sensory integration flow, as described by Mesulam (1998). Gradients were computed using Python (Python version 3.8.10) and the *BrainSpace* library (Vos de Wael et al., 2020) (Python package version 0.1.3). Gradients computed at the regional and vertex levels performed similarly (Vos de Wael et al., 2020).

Average region-level functional connectivity matrices were generated for each individual across the entire cortex (i.e., 384 AICHA brain regions). Consistent with prior work, each region’s top 10% connections were retained, and other elements in the matrix were set to 0 to enforce sparsity (Dong et al., 2021; Margulies et al., 2016). The normalized angle distance between any two rows of a matrix was calculated to obtain a symmetrical similarity matrix. Diffusion map embedding (Coifman & Lafon, 2006; Coifman et al., 2005; Lafon & Lee, 2006) was implemented on the similarity matrix to derive the first gradient. Note that the individual-level gradients were aligned using Procrustes rotation (*N*_iterations_ = 10) to the corresponding group-level gradient. This alignment procedure was used to improve the similarity of the individual-level gradients to those from the literature. Min-max normalization (0–100) was performed at the individual level for the whole brain (Gonzalez Alam et al., 2022).

Gradient asymmetry was then computed for each participant and region. For a given region, gradient asymmetry corresponded to the difference between the normalized gradient value in the left hemisphere minus the gradient values in the right hemisphere. A positive gradient asymmetry value meant a leftward asymmetry; a negative value meant a rightward asymmetry.

### 2.7 Statistical analyses

Statistical analysis was performed using R (R version 4.2.2; R Core Team, 2021). Data wrangling was performed using the R library *dplyr* (R package version 1.0.10; Wickham et al., 2023). Graphs were realized using the R library *ggplot2* (R package version 3.4.2; Wickham, 2016). Brain visualizations were realized using Surf Ice (*NITRC: Surf Ice: Tool/Resource Info*, n.d.), and were made reproducible following guidelines to generate programmatic neuroimaging visualizations (Chopra et al., 2023).

#### 2.7.1 Modeling gradient asymmetry trajectories throughout life

For each region of the Language-and-Memory Network, we used factor-smooth Generalized Additive Mixed Models (GAMMs, as implemented in the R library *gamm4*; R package version 0.2-6; Wood & Scheipl, 2020) to fit a smooth gradient trajectory for age per hemisphere (Roe et al., 2021, 2023) and to assess the smooth interaction between Hemisphere × Age within the clusters (see clusters definition below). Hemisphere was included as a fixed effect, while sex and site were treated as covariates of no interest. A random intercept for each subject was also included. GAMMs leverage smooth functions to model the non-linear trajectories of mean levels across individuals, providing robust estimates that can be applied to cross-sectional and longitudinal cognitive data (Ø. Sørensen et al., 2021). GAMMs were implemented using splines, a series of polynomial functions joined together at specific points, known as knots. The splines allow the smooth function to adapt its shape flexibly to the underlying pattern in the data across the range of the predictor variable. This connection allows for the modeling of complex, non-linear relationships piecewise while maintaining continuity and smoothness across the function. To minimize overfitting, the number of knots was constrained to be low (*k* = 6). The significance of the smooth Hemisphere×Age interaction was assessed by testing for a difference in the smooth term of age between hemispheres. We applied a false discovery rate correction (FDR; Benjamini & Yekutieli, 2001) to control for the number of tests conducted. Lastly, we used the linear predictor matrix of the GAMMs to obtain asymmetry trajectories underlying the interaction Hemisphere×Age and their confidence intervals. These were computed as the difference between zero-centered (i.e., demeaned) hemispheric age trajectories.

#### 2.7.2 Classification of age–asymmetry trajectories

To classify the regions of the Language-and-Memory Network found significant (after applying the FDR correction) according to their functional asymmetry skewness profile (i.e., increasing leftward asymmetry from baseline, decreasing leftward asymmetry, or stabilizing asymmetry with age), we computed a dissimilarity matrix (sum of square differences) between all trajectories. We applied the Partition Around Medoids algorithm (R library *cluster*; R package version 2.1.4; Maechler et al., 2022) to identify clusters of regions sharing identical lifespan trajectories. Clustering solutions from two to seven were considered, and the mean silhouette width determined the optimal solution.

#### 2.7.3 Canonical correlation analysis to assess brain–behavior associations

For each cluster, we assessed the linear relationship between the gradient asymmetry trajectories of the Language-and-Memory Network, their normalized volume, and cognitive language performance using permutation-based Canonical Correlation Analyses (CCA; Wang et al., 2020) inference. CCA is a multivariate statistical method identifying linear combinations of two sets of variables that correlate maximally. CCA reveals modes of joint variation, shedding light on the relationship between cognitive language performance (behavioral set), the lifespan trajectories of sensory integration flow asymmetry, and its underlying anatomy (brain set). The CCA results with a set of *m* mutually uncorrelated (i.e., orthogonal) modes. Each mode captures a unique fraction of the multivariate brain and behavior covariation that is not explained by any of the other *m*−1 modes. To assess statistical significance, we determined the robustness of each estimated CCA mode using permutation testing with 1,000 permutations. This test computes *p*-values to assess the null hypothesis of no correlation between components, adhering to the resampling method developed by Winker et al. (2020). *p*-values were controlled over family-wise error rate (FWER; FWER corrected *p*-values are denoted *p*_FWER_), which is more appropriate than the FDR correction when measuring the significant canonical modes (Winkler et al., 2020).

Before conducting the CCA, we summarized the high-dimensional set of brain variables (gradient and normalized volume asymmetries) using principal component analysis (PCA; Wang et al., 2020). We retained components corresponding to the elbow point in the curve, representing the variance explained by each successive principal component. This was achieved using the R library *PCAtools* (R package version 2.5.15; Blighe & Lun, 2021). These retained principal components were then designated as the brain set for the CCA. Finally, we residualized the two variable sets (brain and behavior sets) to remove the influence of sex, age, and MMSE before executing the CCA.

The CCA has only been realized on the 554 participants of the CamCAN database due to a lack of behavioral data for other participants.

## 3 Results

### 3.1 Evolution of hemispheric gradient asymmetries

We investigated age-related changes in the asymmetry of the functional connectivity architecture asymmetry within the extended Language-and-Memory Network (Fig. 2; Roger et al., 2020) across the adult lifespan using anatomical and resting-state fMRI data acquired at 3T (*n* = 728, aged 18 to 88 years), combining 3 databases (Camcan, Omaha, and Grenoble sample). Demographics are available in the Methods section (Database Demographics).

As described by Labache, Ge, et al. (2023), we took advantage of recent mathematical modeling of the cortex’s functional topography, as Margulies et al. (2016) proposed. First, functional connectivity matrices (384 × 384 AICHA parcels; Joliot et al., 2015) across the full sample were decomposed into components that capture the maximum variance in connectivity. Consistent with prior work (Dong et al., 2021; Margulies et al., 2016), diffusion map embedding (Coifman & Lafon, 2006) was used to reduce the dimensionality of the connectivity data through the nonlinear projection of the voxels into an embedding space. The resulting functional components or manifolds, termed gradients, are ordered by the variance they explain in the initial functional connectivity matrix. The present analysis focused on the first gradient accounting on average for 20% of the total variance in cortical connectivity (respectively, 22% for the sample collected in Omaha, 20% for the CamCAN database, and 19% for the sample collected in Grenoble). In line with prior work (Bernhardt et al., 2022; Hong et al., 2020; Margulies et al., 2016; Mckeown et al., 2020), one end of the principal gradient of connectivity was anchored in unimodal regions, while the other end encompassed broad expanses of the association cortex.

The Language-and-Memory Network corresponds to 37 homotopic regions of interest (Roger et al., 2020) (Fig. 2), either specialized for language (Labache et al., 2019, 2020), episodic memory (Spaniol et al., 2009), or both. Each region is described by its gradient asymmetry value. To identify regions with changing asymmetry across the lifespan, and as described by Roe et al. (2021, 2023), we used a factor-smooth Generalized Additive Mixed Model with Hemisphere × Age (i.e., age-related change in asymmetry) as the effect of interest.

**Fig. 3.**
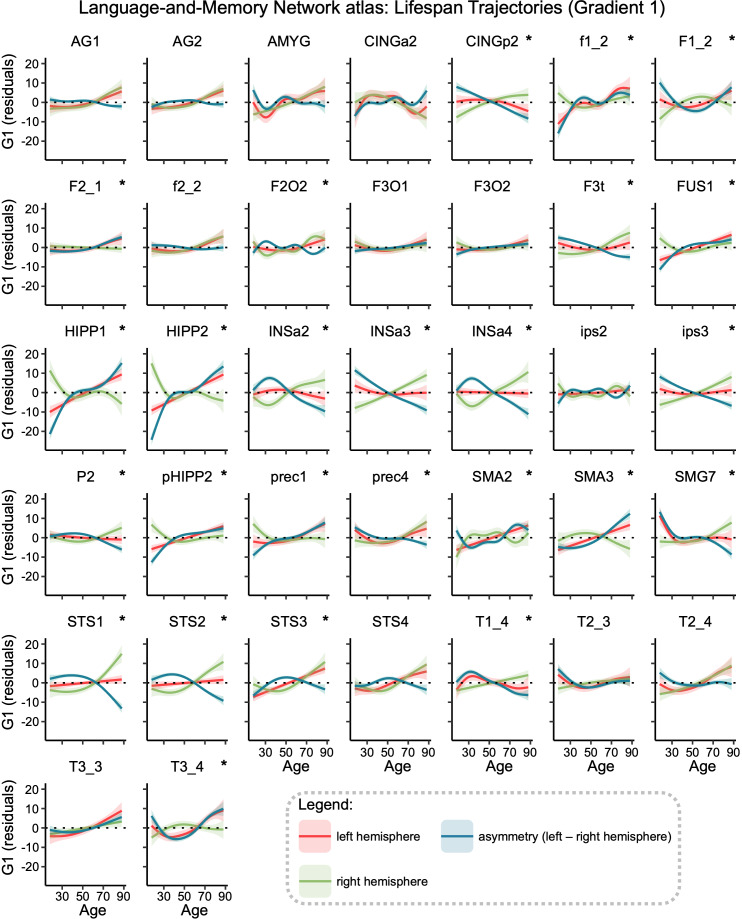
Gradient lifespan trajectories of Language-and-Memory regions. Each region’s graph shows the lifespan trajectory of the left (in red) and the right (in green) hemispheres and their asymmetry (in blue; positive values indicate leftward asymmetries, negative ones indicate rightward asymmetries). Regions are plotted in alphabetical order. Trajectories were fitted using the generalized additive mixed models. Significant regions (*p_FDR_* < 0.05) are marked with a star (*) in the top right corner. Data are residualized for sex, site, and random subject intercepts. Ribbons depict the standard error of the mean. The location of regions is shown in Figure 2. Correspondences between the abbreviations and the full names of a region are given in Supplementary Table S1.

Gradient significant age-related changes in asymmetry were found in 25 of the 37 regions of the Language-and-Memory Network (68% of the Language-and-Memory Network regions, all *p*_FDR_ < 0.024, Fig. 3). On the lateral surface of the temporal lobe, significant regions were localized alongside the superior temporal sulcus (STS1, STS2, STS3), extending to the superior temporal gyrus dorsally (T1_4) and joining the posterior part of the inferior temporal gyrus (T3_4) and ventrally, the fusiform gyrus (FUS4). Advancing toward the parietal lobe, the supramarginal gyrus (SMG7), the inferior parietal gyrus (P2), and the intraparietal sulcus (ips3) also showed significant Hemisphere × Age interactions. On the lateral surface of the left frontal lobe, the regions showing a significant Hemisphere × Age interaction covered the *pars triangularis* part of the inferior frontal gyrus (F3t), as well as the *pars orbitalis* (F2O2), the junction of the middle frontal gyrus (F2_1) with the precentral sulcus (prec1, and prec4). The superior frontal sulcus (f1_2), the medial part of the superior frontal gyrus (F1_2), and the pre-superior motor areas (SMA2 and SMA3) were also part of these areas in the frontal lobe. Three regions were located within the anterior insula (INSa2, INSa3, and INSa4), while three others were located along the hippocampal (HIPP1 and HIPP2) and parahippocampal gyri (pHIPP2). The posterior cingulum (CINGp2) was selected in the posterior medial wall using this approach. The 12 non-significant regions (all *p*_FDR_ > 0.174) were localized in the posterior part of the temporal (STS4, T2_3, T2_4, and T3_3) and the parietal lobes (AG1, AG2, and ips2), the anterior cingulate (CINGa2), the amygdala (AMYG), and the inferior frontal gyrus (F3_O1, F3_O2) and sulcus (f2_2). See Supplementary Table S2 for a description of the asymmetry in early and late life for the 25 regions showing significant age-related changes in gradient asymmetry.

### 3.2 Clustering of asymmetry trajectories

**Fig. 4.**
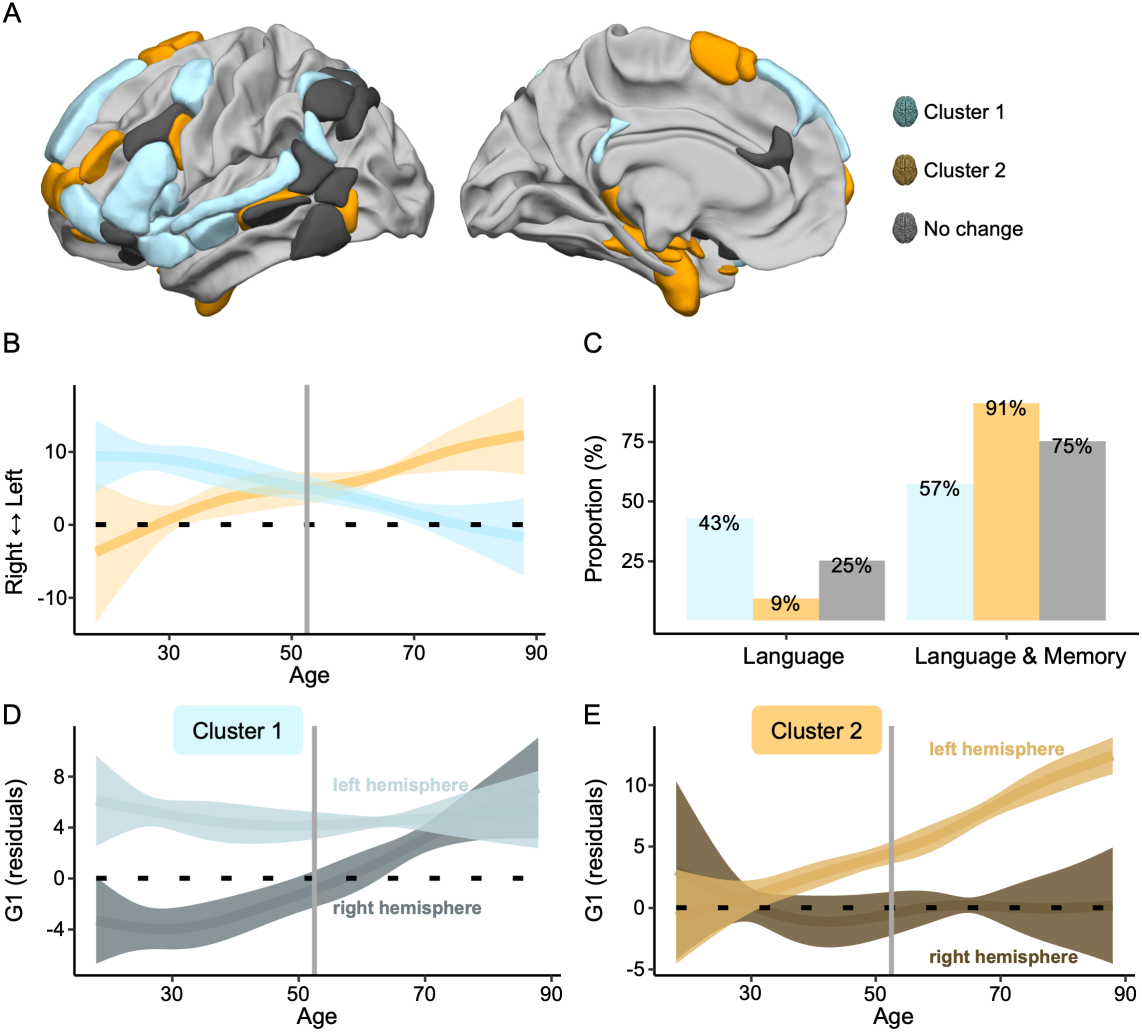
Patterns of language-related neurocognitive trajectories. (A) The 25 Language-and-Memory Network regions associated with the two main clusters of change, categorized according to the *k*-medoids classification applied to the Euclidean distance matrix derived from the age-related curves of asymmetry as modeled by the Generalized Additive Mixed Model. Cluster 1, in blue, changes from left-sided dominant to bilateral. Cluster 2, in orange, changes from a bilateral organization to a left-side dominance. See Figure 2 and Supplementary Table S1 for a description of the regions. (B) Average trajectory curves of the 1^st^ gradient asymmetries from 18 to 88 years old. The two main patterns of inverse changes (Cluster 1 and Cluster 2) with age. The vertical line represents the intersection point between Cluster 1 and Cluster 2: 52.55 years old, that is, the age at which the 1^st^ gradient asymmetry trends reverse. Ribbons depict the standard deviation. (C) The proportion of each cluster depends on the underlying cognitive processes: language or language and memory. (D–E) Modeling of the average estimated 1^st^ gradient parameter for each hemisphere (left and right) across ages for Language-and-Memory Network regions belonging to Cluster 1 (D) and Cluster 2 (E). Ribbons depict the standard deviation. The bilateralization of Cluster 1 with age is due to an increase of the 1^st^ gradient values in the right hemisphere, while the left hemisphere remains stable. The left-sided specialization of Cluster 2 with age is due to an increase of the 1^st^ gradient values in the left hemisphere, while the right hemisphere remains stable. This dual mechanism is mediated by an overspecialization of the contralateral hemisphere with age, characterized by an increased capacity to integrate high-level Language-and-Memory Network information.

To investigate the asymmetry trajectories associated with the Hemisphere×Age interaction, we conducted clustering on the 25 significant regions within the Language-and-Memory Network to pinpoint areas displaying similar patterns of gradient asymmetry changes throughout adulthood (Fig. 3). The Partition Around Medoids algorithm identified two optimal partitions based on the mean silhouette width of 0.73. Including the regions that did not exhibit significant changes in gradient asymmetries over the lifespan, the Language-and-Memory Network regions are grouped into three distinct clusters (Fig. 4A).

The first cluster, highlighted in light blue in Figure 4 and referenced similarly throughout the paper, comprised regions that showed an average increase in their gradient values in the right hemisphere (Fig. 4D). These regions transitioned to a slightly rightward asymmetrical state with aging (*smooth_88 yo_* = -1.72), whereas they exhibited leftward asymmetry in earlier life stages (*smooth_18 yo_* = 9.40, negative slope from positive intercept, Fig. 4B). The right hemisphere heteromodality increased significantly with aging, while the left hemisphere capacity remained stable. Within this cluster, 43% of the regions were dedicated to processing language, while 57% were multimodal, handling language and memory functions (Fig. 4C). Cluster 1 regions are mapped onto the frontal, parietal, temporal, limbic cortices, and insula.

The second cluster, highlighted in light orange in Figure 4 and referenced similarly throughout the paper, comprised regions that showed an average increase in their gradient values in the left hemisphere (Fig. 4E). These regions transitioned to a leftward asymmetry state with aging (*smooth_88 yo_* = 12.23), whereas they exhibited rightward asymmetry organization in earlier life stages (*smooth_18 yo_* = -3.77, positive slope from negative intercept, Fig. 4B). The left hemisphere heteromodal specialization increased significantly with aging, while the right hemisphere capacity remained stable. Within this cluster, 9% of the regions were dedicated to processing language, while 91% were multimodal, handling language and memory functions (Fig. 4C). Cluster 2 regions are mapped onto the frontal, temporal, and limbic cortices.

The last cluster (gray in Fig. 4), named “No change,” regrouped the 12 non-significant regions that showed no significant changes in their hemispheric asymmetries throughout the lifespan. This cluster encompasses 25% of regions exclusively associated with language function and 75% of the regions involved in language and memory processes.

The trajectories of Clusters 1 and 2 indicated that the asymmetry switch occurred at 52.6 years (Fig. 4B). From this age onward, Cluster 2, which mainly encompasses multimodal regions, became the dominant leftward asymmetrical cluster. Its heteromodality in later life surpassed the early life heteromodality of Cluster 1. Meanwhile, Cluster 1 continued its decline toward a symmetrical organization of information integration.

### 3.3 Multimodal brain–cognition association change analysis

Finally, we examined the extent to which changes in functional asymmetries among the two clusters are associated with individual differences in language-related cognitive performance across the adult lifespan. To gain a comprehensive understanding, our analysis also incorporated the normalized volume of each region within the identified clusters. This approach allowed us to identify a tripartite relationship connecting anatomy, macroscale functional brain organization, and cognitive performance across different ages. To achieve this, we used permutation-based Canonical Correlation Analysis (CCA) inference (Wang et al., 2020). CCA reveals modes of joint variation between two sets of variables, resulting in a set of mutually uncorrelated modes. Each mode captures a portion of the multivariate brain and behavior covariation. The CCA was conducted between a set of brain variables (including gradients and normalized volumes) and a set of cognitive variables evaluating language performance (including naming and tip of the tongue for language production and accuracy and reaction time in language comprehension). Language skill assessments are described in the Methods section (Cognitive Assessment of Participants). Prior to conducting CCA, we summarized the high-dimensional set of brain variables using principal component analysis (Wang et al., 2020) (PCA). The CCA has been performed on the 554 participants of the CamCAN database only due to a lack of behavioral data for other participants.

**Fig. 5.**
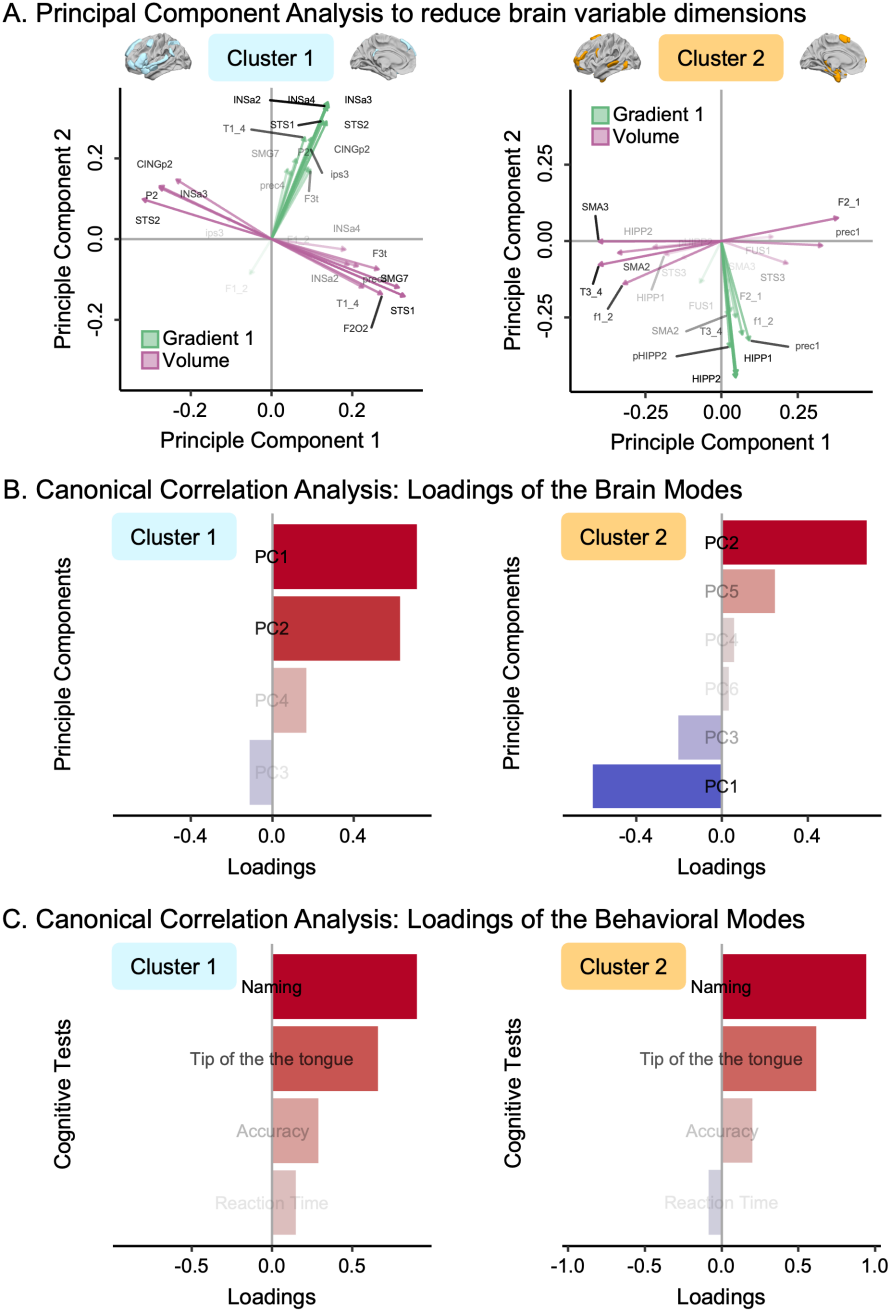
Brain–behavior association using canonical correlation analysis. (A) Biplot of the principal component analysis of the regions belonging to Cluster 1 (*n* = 14, on the left) and Cluster 2 (*n* = 11, on the right). Each region was characterized by its asymmetry values of the 1^st^ gradient and normalized volume. The two principal components of Cluster 1 explained 38.89% of the total variance (*Principal Component 1* = 26.74%, *Principal Component 2* = 12.15%). The two principal components of Cluster 2 explained 33.34% of the total variance (*Principal Component 1* = 20.47%, *Principal Component 2* = 12.87%). For Cluster 1, the 1^st^ principal component opposed the volume asymmetries of the dorsal language pathway regions to the ventral semantic pathway regions. The 2^nd^ component opposed the symmetries of the 1^st^ gradient to the symmetries of the normalized volume. For Cluster 2, the 1^st^ principal component opposed the asymmetry of mesial regions *versus* the volume asymmetry of lateral regions. The 2^sd^ component coded for the symmetry of the 1^st^ gradient, specifically, the symmetry of the temporo-mesial memory-related regions: a larger value meant a larger symmetry. (B–C) Overview of the canonical correlation analysis first modes. Only data from participants with all scores on the selected language indicators were included in the analysis (*n* = 554; CamCAN cohort only). Sex, age, and general cognitive status (MMSE) were entered as covariates. (B) First mode for brain variables. For Cluster 1, the brain mode explained 38% of the variance. It is saturated by the first two components of the principal component analysis, mixing the multimodal biomarkers included in the analysis (1^st^ gradient and normalized volume). For Cluster 2, the brain mode explained 24% of the variance. It is saturated by the first two components of the principal component analysis. (C) First mode of behavioral variables. For Clusters 1 and 2, the behavioral mode explained 39% of the variance and was saturated by the language production tasks involving lexical access and retrieval: naming and tip of the tongue. Results for Cluster 1 are framed in light blue. Results for Cluster 2 are framed in orange.

**Cluster 1 –** We first conducted a PCA on the brain set variables (gradient and normalized volume asymmetries) from the first cluster (Fig. 4A). This analysis indicated that the 28 variables could be condensed into 4 principal components, accounting for 49.79% of the total variance in the brain set. The first component alone explained 26.75% of the total variance and opposed the volume asymmetries of the dorsal language pathway regions to those of the ventral pathway regions (Fig. 5A, left column). Positive loadings then indicated a leftward asymmetry of the dorsal pathway, while negative loadings indicated a rightward asymmetry of the ventral pathway. The second component alone explained 12.15% of the total variance. It opposed the volume asymmetries of the dorsal language pathway regions to those of the ventral pathway regions and the asymmetries of the first gradient (Fig. 5A, left column). Positive loadings then indicated a rightward asymmetry of the volume of the dorsal pathway regions and a leftward asymmetry of the ventral pathway as well as the gradient values. At the same time, negative loadings indicated the opposite pattern.

**Fig. 6.**
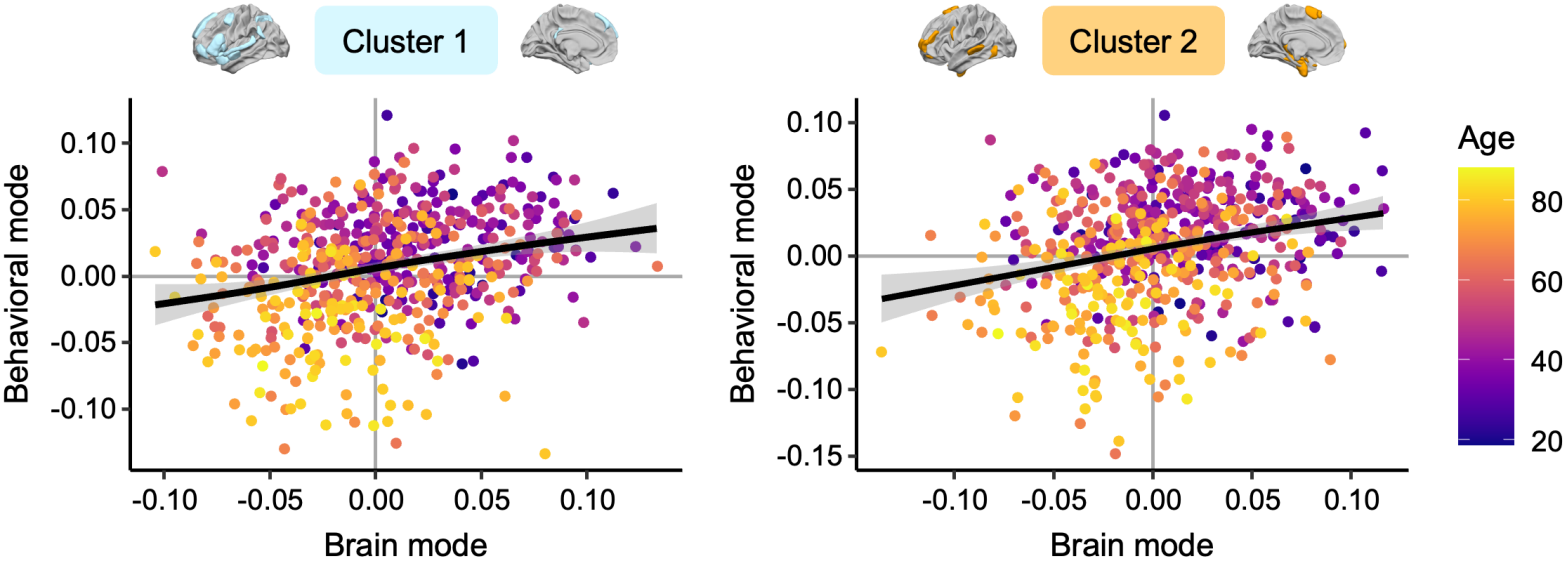
Relationship between changes in inter-hemispheric balances and their behavioral implications in a multimodal perspective. The first brain and behavioral modes were significantly correlated for both clusters: *r* = 0.28, *p* < 1.10^-3^. The significance of correlations between modes was assessed using permutation testing (*n* = 1000). Color code for age.

The multimodal canonical correlation analysis on the first cluster, which incorporated four brain metrics (principal components) and four behavioral metrics, revealed a single significant canonical correlation linking anatomy, function, and behavior (*p*_FWER_ < 1 × 10^-3^). We investigated the CCA loadings for the brain set of variables, which represent the contribution of each PCA component to the significant canonical correlation and are referred to as the “brain mode” hereafter. This brain mode accounted for 37.58% of the variance and primarily reflected the first and second PCA components of the brain data set (Fig. 5B, left column). Positive values of the brain mode were associated with positive loading values for both the first and second principal components. Specifically, these positive values in the brain mode indicated a leftward asymmetry for all regions regarding gradient and normalized volume in the dorsal language pathway regions. Conversely, they represented a rightward asymmetry in the ventral pathway regions. We analyzed the CCA loadings for the behavioral set of variables, which represent the contribution of each behavioral language test to the significant canonical correlation and are referred to as the “behavioral mode” hereafter. The behavioral mode accounted for 39.47% of the variance and primarily reflected the naming and tip of the tongue tests (Fig. 5C, left column). Positive values of the behavioral mode were associated with better performances in language production. The correlation between the brain and behavioral modes was 0.28, as depicted in Figure 6 (left panel). Improved language production abilities were linked to a leftward asymmetry of the gradient value within the Language-and-Memory Network regions of the first cluster, a leftward asymmetry of the normalized volume for the dorsal language pathway regions, and a rightward asymmetry for the ventral language pathway regions.

**Cluster 2 –** The principal components analysis on the brain set variables (22 variables, gradient, and normalized volume asymmetries) for the second cluster (Fig. 4A) resulted in 6 principal components. Together, these principal components explained 59.35% of the total variance in the brain set. The first component alone explained 20.48% of the total variance and opposed the volume asymmetries of the mesial regions to those of the lateral side (Fig. 5A, right column). Positive loadings then indicated a rightward asymmetry of the normalized volume of the mesial regions and a leftward asymmetry of the lateral regions. Negative loadings indicated the opposite pattern. The second component alone explained 12.86% of the total variance and captured the asymmetry of the gradient, specifically, the asymmetry of the temporo-mesial memory-related regions (Fig. 5A, right column). Positive loadings indicated a rightward asymmetry of the gradient, while negative loadings indicated a leftward asymmetry.

The multimodal canonical correlation analysis on the second cluster, which incorporated six brain metrics (principal components) and four behavioral metrics, revealed a single significant canonical correlation linking anatomy, function, and behavior (*p*_FWER_ < 1 × 10^-3^). This brain mode accounted for 23.61% of the variance and opposed the second component of the brain data set to the first one (Fig. 5B, right column). Positive values of the brain mode were associated with positive loading values for the second component and negative values for the first component. A positive brain mode value meant a leftward asymmetry of the normalized volume of the mesial regions, a rightward asymmetry of the lateral regions, and a rightward asymmetry of the gradient. The behavioral mode accounted for 39.04% of the variance and, similarly to Cluster 1, primarily reflected the naming and tip of the tongue tests (Fig. 5C, right column). The correlation between the brain and behavioral modes was 0.28, as depicted in Figure 6 (right panel). Improved language production abilities were linked to a rightward asymmetry of the gradient value within the temporo-mesial memory-related regions, a leftward asymmetry of the normalized volume of the mesial regions, and a rightward asymmetry of the normalized volume of the lateral regions.

## 4 Discussion

Our study uncovers that functional asymmetry in the integration of high-level information plays a pivotal role in the neural mechanisms underlying language processing and capabilities. Trajectory modeling across the lifespan revealed shifts in hemispheric dominance, underscoring the dynamic nature of functional lateralization. These changes in asymmetry are associated with the language production challenges commonly seen in typical aging, disputing the notion that increased engagement of the contralateral hemisphere in older adults serves a compensatory role. Instead, our findings align with the brain maintenance theory, highlighting the importance of preserving a youthful functional brain state for optimal cognitive performance as individuals age. This study paves the way for further exploration into the dynamic processes by which the brain and cognition adapt throughout the aging process.

We found that this dual mechanism of the Language-and-Memory Network neurofunctional imbalance in integrating complex, high-level information begins after age 50 years and intensifies over time (Fig. 4B). These findings are consistent with previous functional studies showing significant transitions in middle age (Hennessee et al., 2022). They also align with the onset of structural changes observed in healthy older adults regarding cortical thickness asymmetry, showing an accelerated loss of asymmetry after midlife (Fjell et al., 2010; Roe et al., 2021; Vidal-Piñeiro et al., 2019). The reduction in structural asymmetry is notably significant in higher-order cortex and heteromodal regions, which may account for the extensive reorganization observed in the functional organization of the Language-and-Memory Network regions. None of these changes in asymmetry contributed to maintaining language performance with age and were, instead, linked to poorer performance. For Cluster 1 and Cluster 2, the pattern observed in young adults was related to more efficient language production (Fig. 6), underlining the importance of specialization at all ages for effective interhemispheric cooperation. Consequently, the changes do not support the hypothesis of a compensatory phenomenon (Cabeza et al., 2018), preserving language performance with age. On the contrary, it aligns with the dedifferentiation theory of aging (Li et al., 2009; Morcom & Friston, 2012; Morcom & Henson, 2018; Reuter-Lorenz & Lustig, 2005) and the brain maintenance theory (Nyberg, 2017; Nyberg et al., 2012), suggesting that maintaining a (functional) youthful brain state is essential to cognitive preservation as individuals age. These findings further underscore Roe and colleagues’ insights in their recent investigation of age-related shifts in functional asymmetry during memory retrieval (Roe et al., 2020).

Our results suggest that changes in language lateralization across adulthood are not isolated phenomena but are deeply embedded in a broader reorganization of the brain’s functional architecture. This aligns with the concept of “complementary lateralization,” where specialization for language in the left hemisphere is counterbalanced by the right hemisphere’s engagement in non-verbal, high-level functions such as visuospatial processing (Badzakova-Trajkov et al., 2010; Cai et al., 2013; Cochet, 2016; Labache et al., 2024; Serrien & O’Regan, 2022; Zago et al., 2016). Our observation that left hemisphere regions in Cluster 2 gain functional specialization with age while right hemisphere-dominated Cluster 1 becomes more bilateral supports this interdependent view. It also echoes prior findings that reduced language lateralization is linked to diminished performance in both linguistic and non-linguistic domains (Mellet et al., 2014). This may reflect a breakdown in the fine-tuned functional segregation necessary for optimal cognitive efficiency. Furthermore, control networks—crucial for managing interactions across these cognitive domains—undergo significant reconfigurations with age (Baciu et al., 2021; Betzel et al., 2014; Doucet et al., 2021; He et al., 2013; Mowinckel et al., 2012; Roger et al., 2022). Our findings may thus reflect an age-related shift in the equilibrium of inter-network coordination, particularly along the cortical gradient that spans from heteromodal to unimodal systems (Gonzalez Alam et al., 2022). These large-scale reconfigurations are likely constrained by underlying molecular and cellular asymmetries—such as lateralized neurotransmitter receptor distributions and mitochondrial profiles—that support functional hemispheric specialization (Labache et al., 2025). Investigating how these network-level rebalancings relate to functional specialization in aging may yield key insights into both the plasticity and vulnerability of the aging brain.

While functional connectivity gradients offer a parsimonious framework to capture macroscale cortical organization and have shown strong correspondence with known functional hierarchies (Bethlehem et al., 2020; Margulies et al., 2016), they should be interpreted cautiously as non-causal indicators of cognitive architecture. As recently pointed out (Herbet & Duffau, 2020), functional connectivity lacks the causal specificity of techniques such as direct electrostimulation or lesion-based approaches, and may not straightforwardly reflect the underlying cognitive processes. In this context, our use of the principal gradient aims to describe large-scale shifts in functional integration across hemispheres rather than to assign direct cognitive functions to specific regions. These gradient-derived metrics are best viewed as descriptive tools that complement (Labache, Ge, et al., 2023), rather than replace, task-based or causal mapping approaches.

The human brain typically exhibits marked left–right asymmetries, especially in perisylvian regions involved in language. While genetics play a role in shaping these asymmetries, their heritability appears limited (under 30% in adults; (Kong et al., 2018; Sha et al., 2021)), suggesting a major influence of environmental and experiential factors. Our results, showing a shift toward greater leftward asymmetry in multimodal language-and-memory regions (Cluster 2) and a bilateralization of classically left-lateralized regions (Cluster 1), may reflect such long-term environmental shaping, especially from midlife onward. This supports a two-phase developmental model: an early, genetically guided trajectory followed by a prolonged experience-sensitive period in heteromodal regions (Dong et al., 2024; Labache, Ge, et al., 2023). These age-related shifts in asymmetry may thus be modulated by midlife experiences, lifestyle, and sensory inputs, particularly hearing. Recent evidence, however, suggests that the relationship between hearing loss and dementia may be indirect, potentially mediated by reduced social engagement rather than representing a direct causal link (Ishak et al., 2025; Sarant et al., 2023). Age-related sensory degradation remains a plausible contributor to cortical reorganization, as supported by observed functional changes in the auditory cortex during aging (Huang et al., 2023; Profant et al., 2015; Schulte et al., 2020). Sensory degradation, such as age-related hearing loss, is known to drive neural reconfigurations (Huang et al., 2023; Schulte et al., 2020), and has been identified as a major modifiable risk factor for dementia (Livingston et al., 2020). Given the coupling between sensory input and functional asymmetry (Hesling et al., 2019; Hugdahl & Westerhausen, 2016; Van der Haegen et al., 2016), our findings underscore the need to investigate how bottom-up sensory factors might interact with age-related cortical reorganization, potentially contributing to the dual mechanism of hemispheric change we report.

Several methodological considerations and potential biases require discussion. Our study statistically controlled for gender, and prior work has highlighted gender-based disparities in language-related functional connectivity (Roger et al., 2022) and hemispheric asymmetries (Liang et al., 2021). However, inter-individual variability in network organization may exceed these group-level effects. Future research should, therefore, adopt subject-specific, data-driven approaches to identify language networks, as advocated by Fedorenko et al. (2010), to account for heterogeneity in lateralization patterns, rather than assuming a uniform network architecture across individuals. This will be particularly important in aging populations, where both individual variability and sex-related effects may interact and shape the trajectories of hemispheric specialization. Moreover, our study predominantly included participants from WEIRD (Western, Educated, Industrialized, Rich, and Democratic) societies. Considering that most of the global population does not fit within this category (Henrich et al., 2010), it would be beneficial to replicate these findings in more diverse populations, considering the importance of cultural diversity in research. Resting-state functional MRI has gained popularity due to its strong association with task-based fMRI activations (Cole et al., 2014, 2016) and ease of acquisition, rendering it a valuable proxy for capturing functional neuronal processes. Nevertheless, the strength of hemispheric specialization for language depends on multiple factors, particularly the nature of the task (Bradshaw et al., 2017; Labache et al., 2019). Hence, conducting an additional study encompassing a diverse array of language-related functional tasks is essential to validate the consistency of the trends observed in our resting-state functional data. Open fMRI databases dedicated to language, such as InLang (Roger et al., 2022), could facilitate such investigations. However, the databases available to date only sometimes include a wide age range, which could limit insights into older adults. Finally, longitudinal data are imperative for providing conclusive evidence regarding evolutionary trajectories throughout the lifespan and their cognitive implications. The STAC-r model (revised Scaffolding Theory of Aging and Cognition model) emphasizes the importance of examining cognitive changes within individuals (Reuter-Lorenz & Park, 2014). This approach helps distinguish between mechanisms that maintain brain integrity and compensatory processes. Both mechanisms are crucial for preserving cognition in older adults, as noted by Reuter-Lorenz and Park (2014). However, the current scarcity of extensive longitudinal cohorts, spanning both older and younger adults, hinders the identification of features predictive of future brain function and cognitive preservation (Doucet et al., 2022). It would also be important to extend the study to cohorts with mild cognitive impairment (MCI) and related conditions, which is crucial for assessing the specificity of the observed effects and discerning trends across different conditions.

In summary, this study provides novel evidence that age-related changes in hemispheric specialization for language follow a dual mechanism: a shift toward increased leftward asymmetry in multimodal language-and-memory regions, and a simultaneous bilateralization of classically left-lateralized regions. By integrating functional gradients, structural asymmetries, and behavioral measures across a large adult lifespan sample, our findings reveal how aging reshapes the brain’s intrinsic language architecture in ways that are linked to cognitive performance. These insights refine current models of cognitive aging, highlight the importance of midlife transitions, and lay groundwork for personalized approaches to language-based interventions in aging populations.

## Data and Code Availability

The CamCAN dataset is available upon request through the Cambridge Centre for Ageing and Neuroscience (CamCAN) website (Shafto et al., 2014; Taylor et al., 2017): https://www.cam-can.org. Access requires completion of a data access application, which includes agreeing to the CamCAN data usage policies and providing a brief description of the intended research purpose. Further details can be found at: https://camcan-archive.mrc-cbu.cam.ac.uk/dataaccess/. The Omaha dataset (NE, USA) includes de-identified data and is available upon request to G. E. Doucet (gaelle.doucet@boystown.org). The Grenoble dataset (France) is available upon request to M. Baciu (monica.baciu@univ-grenoble-alpes.fr). Access requires prior approval from the local institutional review board (IRB), completion of a Data Use Agreement, and submission of a brief description of the intended use, along with evidence of ethical approval.

The atlas and the code used to produce the results and visualizations can be found here (Labache, Roger, et al., 2023): https://github.com/loiclabache/RogerLabache_2023_LanguAging.

## Author Contributions

Elise Roger: Conceptualization, Data curation, Formal analysis, Investigation, Methodology, Software, Validation, Visualization, Writing—original draft, Writing—review & editing. Loïc Labache: Conceptualization, Data curation, Formal analysis, Investigation, Methodology, Resources, Software, Supervision, Validation, Visualization, Writing—original draft, Writing—review & editing. Noah Hamlin: Data curation, Investigation. Jordanna Kruse: Data curation, Investigation. Monica Baciu: Conceptualization, Data curation, Funding acquisition, Investigation, Methodology, Resources, Supervision, Writing—review & editing. Gaelle E. Doucet: Conceptualization, Data curation, Funding acquisition, Investigation, Supervision, Writing—review & editing.

## Funding

E.R. received funding support from the Canadian Institutes of Health Research (CIHR), the “Fonds de Recherche du Québec—Santé” (FRQS), and the AGE-WELL Canadian network. This work was supported by the grant NeuroCoG IDEX UGA in the framework of the “Investissements d’avenir” program (ANR-15-IDEX-02 to M.B.), by the French program “AAP GENERIQUE 2017” run by the “Agence Nationale pour la Recherche” (ANR-17-CE28–0015-01 to M.B.) and by the Institut Universitaire de France (M.B.), as well as by the following awards to G.E.D.: The National Institutes of Health (P20GM144641, R03AG064001). The content is solely the responsibility of the authors and does not necessarily represent the official views of the National Institutes of Health.

## Declaration of Competing Interest

The authors declare no actual or potential conflict of interest.

## Supplementary Material

Supplementary Material
