# Supplementary material for: When age tips the balance: A dual mechanism affecting hemispheric specialization for language
Source: Imaging Neurosci (Camb). 2025 Jul 2;3:IMAG.a.63. doi: 10.1162/IMAG.a.63 (PMC12330839; doi:10.1162/IMAG.a.63)
Supplement: Supplementary Material [file IMAG.a.63_supp.pdf]

Supplementary Materials

When Age Tips the Balance: a Dual Mechanism Affecting Hemispheric Specialization for Language

Elise Roger[a, b, c *, †], Loïc Labache[d, e, *, †], Noah Hamlin[f, g], Jordanna Kruse[f, g], Monica Baciu[c], Gaelle E. Doucet[f, g, h]

[a] Institut Universitaire de Gériatrie de Montréal, Communication and Aging Lab, Montreal, Quebec, Canada
[b] Faculty of Medicine, University of Montreal, Montreal, Quebec, Canada
[c] Univ. Grenoble Alpes, Univ. Savoie Mont Blanc, CNRS, LPNC, 38000 Grenoble, France
[d] Department of Psychology, Yale University, New Haven, CT, 06520, US
[e] Department of Psychiatry, Brain Health Institute, Rutgers University, Piscataway, NJ, 08854, US
[f] Institute for Human Neuroscience, Boys Town National Research Hospital, Omaha, NE, 68010, US
[g] Department of Pharmacology and Neuroscience, Creighton University School of Medicine, Omaha, NE, 68178, US
[h] Center for Pediatric Brain Health, Boys Town National Research Hospital, Omaha, NE, 68178, US

* equal contribution (first author); these authors contributed equally to this study and can both list themselves as first author in their CVs
† Corresponding authors: Loïc Labache (loic.labache@yale.edu), and Elise Roger (elise.roger@umontreal.ca)

1. Comparative tables of database acquisition parameters

MRI scanner

CamCAN: 3T Siemens TIM Trio scanner with a 32-channel head coil
Omaha: 3T Siemens Prisma scanner with a 64-channel head coil
Grenoble: 3T Philips Achieva TX scanner with a 32-channel head coil

Scan type: Structural MRI scans (T1-weighted)

Cohort	Sequence	TR (*ms*)	TE (*ms*)	Flip angle (°)	FOV (*mm*)	Voxel size (*mm*)	Other
CamCAN	MPRAGE	2250	2.99	9	256 × 240 × 192	1 × 1 × 1	GRAPPA: 2; TI: 900 *ms*
Omaha	MPRAGE	2400	2.22	8	256 × 256 x 256	0.8 × 0.8 × 0.8	GRAPPA: 2; TI: 1000 *ms*
Grenoble	MPRAGE	2100	2.36	8	256 × 240 × 160	0.8 × 0.8 × 0.8	GRAPPA: 2; TI: 1000 *ms*

Notes. TR = repetition time; TE = echo time; FOV = field of view; MPRAGE = magnetization prepared gradient echo; GRAPPA = GeneRalized Autocalibrating Partial Parallel Acquisition; TI = inversion time.

Scan type: Functional MRI scans (resting-state)

Cohort	Sequence	TR (*ms*)	TE (*ms*)	Flip angle (°)	FOV (*mm*)	Voxel size (*mm*)	Volumes (*N*)	Slices (*N*)	Duration	Task
CamCAN	EPI	1970	30	78	192 × 192	3 × 3 × 4.44	261	32	8min 40s	Rest with eyes closed
Omaha	Multi-band EPI	800	37	78	192 × 192	2 × 2 × 2	460	72	11min 83s	Rest with eyes on a fixation cross
Grenoble	EPI	2000	30	75	192 × 192	2 × 2 × 2	400	36	13min 20s	Rest with eyes on a fixation cross

Notes. TR = repetition time; TE = echo time; FOV = field of view; EPI = T2*-weighted gradient echo planar image.

2. List of the Language-and-Memory network atlas regions

Abbreviation	Region	Function	MNI coordinates (left)			MNI coordinates (right)		
			X (*mm*)	Y (*mm*)	Z (*mm*)	X (*mm*)	Y (*mm*)	Z (*mm*)
AG1	Angular Gyrus (1)	M	-48	-57	44	51	-52	43
AG2	Angular Gyrus (2)	LM	-38	-70	39	45	-62	36
AMYG	Amygdala (1)	M	-22	0	-12	21	2	-12
CINGa2	Anterior Cingulate Gyrus (2)	M	-7	34	22	7	33	23
CINGp2	Posterior Cingulate Gyrus (2)	M	-4	-39	27	8	-43	31
f1_2	superior frontal sulcus (2)	M	-27	56	1	28	56	7
f2_2	inferior frontal sulcus (2)	LM	-43	15	29	44	19	28
F1_2	Superior Frontal Gyrus (2)	L	-12	46	41	12	45	42
F2_1	Middle Frontal Gyrus (1)	M	-40	41	20	41	44	13
F2O2	Middle Frontal Gyrus: Pars Orbitalis (2)	M	-41	49	-5	40	50	-4
F3O1	Inferior Frontal Gyrus: Pars Orbitalis (1)	L	-42	31	-17	44	33	-14
F3O2	Inferior Frontal Gyrus: Pars Orbitalis (2)	M	-21	23	-21	21	22	-20
F3t	Inferior Frontal Gyrus: Pars Triangularis (1)	L	-49	26	5	50	29	5
FUS1	Fusiform Gyrus (1)	M	-32	-9	-34	32	-8	-35
HIPP1	Hippocampal Gyrus (1)	M	-30	-7	-19	30	-5	-18
HIPP2	Hippocampal Gyrus (2)	M	-25	-32	-3	25	-31	-2
INSa2	Anterior Insula (2)	LM	-34	17	-13	35	18	-13
INSa3	Anterior Insula (3)	LM	-34	24	1	37	24	0
INSa4	Anterior Insula (4)	M	-41	15	3	41	15	4
ips2	intraparietal sulcus (2)	M	-34	-58	46	37	-52	48
ips3	intraparietal sulcus (3)	M	-27	-60	44	26	-62	46
P2	Inferior Parietal Gyrus (1)	M	-45	-53	50	43	-53	48
pHIPP2	Parahippocampal Gyrus (2)	M	-28	-27	-19	29	-25	-19
prec1	precentral sulcus (1)	M	-50	6	26	50	10	24
prec4	precentral sulcus (4)	LM	-42	1	50	44	1	48
SMA2	Supplementary Motor Area (2)	L	-11	18	63	11	18	63
SMA3	Supplementary Motor Area (3)	LM	-7	8	66	6	10	66
SMG7	Supramarginal Gyrus (7)	L	-55	-52	26	55	-46	33
STS1	superior temporal sulcus (1)	L	-50	14	-22	52	13	-26
STS2	superior temporal sulcus (2)	L	-55	-7	-13	54	-2	-15
STS3	superior temporal sulcus (3)	LM	-55	-33	-2	53	-32	0
STS4	superior temporal sulcus (4)	L	-57	-48	13	55	-46	15
T1_4	Superior Temporal Gyrus (4)	L	-59	-23	4	60	-20	2
T2_3	Middle Temporal Gyrus (3)	LM	-61	-35	-5	62	-31	-5
T2_4	Middle Temporal Gyrus (4)	L	-53	-59	7	57	-53	3
T3_3	Inferior Temporal Gyrus (3)	M	-56	-53	-14	57	-46	-14
T3_4	Inferior Temporal Gyrus (4)	M	-50	-61	-8	54	-58	-11

Supplementary Table 1. List of the Language-and-Memory network atlas regions. Note: L=language; LM=language and memory; M=memory; MNI coordinates, in the left and right hemisphere, of regions (*X*, *Y*, *Z*) in mm; Total regions=74 (37 in each hemisphere).

3. Regional asymmetry trajectories in areas showing a significant age-related effect on functional gradients

Function	Region Abbreviation	Early Life Asymmetry	Late Life Asymmetry
M	CINGp2	Left	Right
M	f1_2	Right	Left
L	F1_2	Left	Left
M	F2_1	Bilateral	Left
M	F2O2	Left	Bilateral
L	F3t	Left	Right
M	FUS1	Right	Left
M	HIPP1	Right	Left
M	HIPP2	Right	Left
LM	INSa2	Bilateral	Right
LM	INSa3	Left	Right
M	INSa4	Bilateral	Right
M	ips3	Left	Right
M	P2	Bilateral	Right
M	pHIPP2	Right	Left
M	prec1	Right	Left
LM	prec4	Left	Right
L	SMA2	Left	Left
LM	SMA3	Right	Left
L	SMG7	Left	Right
L	STS1	Left	Right
L	STS2	Bilateral	Right
LM	STS3	Right	Right
L	T1_4	Bilateral	Right
M	T3_4	Left	Left

Supplementary Table 2. Regional asymmetry trajectories in areas showing a significant age-related effect on functional gradients. Note: L=language; LM=language and memory; M=memory.
